# Supplementary material for: Antibody-mediated NK cell activation as a correlate of immunity against influenza infection
Source: Nat Commun. 2023 Aug 24;14:5170. doi: 10.1038/s41467-023-40699-8 (PMC10449820; doi:10.1038/s41467-023-40699-8)
Supplement: Supplementary file 1 — Supplementary Information [file 41467_2023_40699_MOESM1_ESM.pdf]

Antibody-mediated NK cell activation as a correlate of immunity against influenza infection

Carolyn M. Boudreau[1,2], John S. Burke IV[1], Ashraf S. Yousif[1], Maya Sangesland[1,2], Sandra Jastrzebski[3], Chris Verschoor[4], George Kuchel[3], Daniel Lingwood[1], Harry Kleanthous[5], Iris De Bruijn[6], Victoria Landolfi[7], Saranya Sridhar*[7], Galit Alter*[1]

[1]Ragon Institute of MGH, MIT, and Harvard; Cambridge, MA, 02129, USA
[2]PhD Program in Virology, Division of Medical Sciences, Harvard University; Boston, MA, 02115, USA
[3]Center on Aging, UCONN Health Center; Farmington, CT 06030, USA
[4]Department of Pathology and Molecular Medicine, Faculty of Health Sciences, McMaster University; Hamilton, ON, L8S 4L8, Canada
[5]SK Bioscience; Cambridge, MA, 02141, USA
[6]Sanofi-Pasteur, Inc.; Marcy-l'Étoile, France
[7]Sanofi-Pasteur, Inc.; Cambridge, MA, 02129, USA
*contributed equally

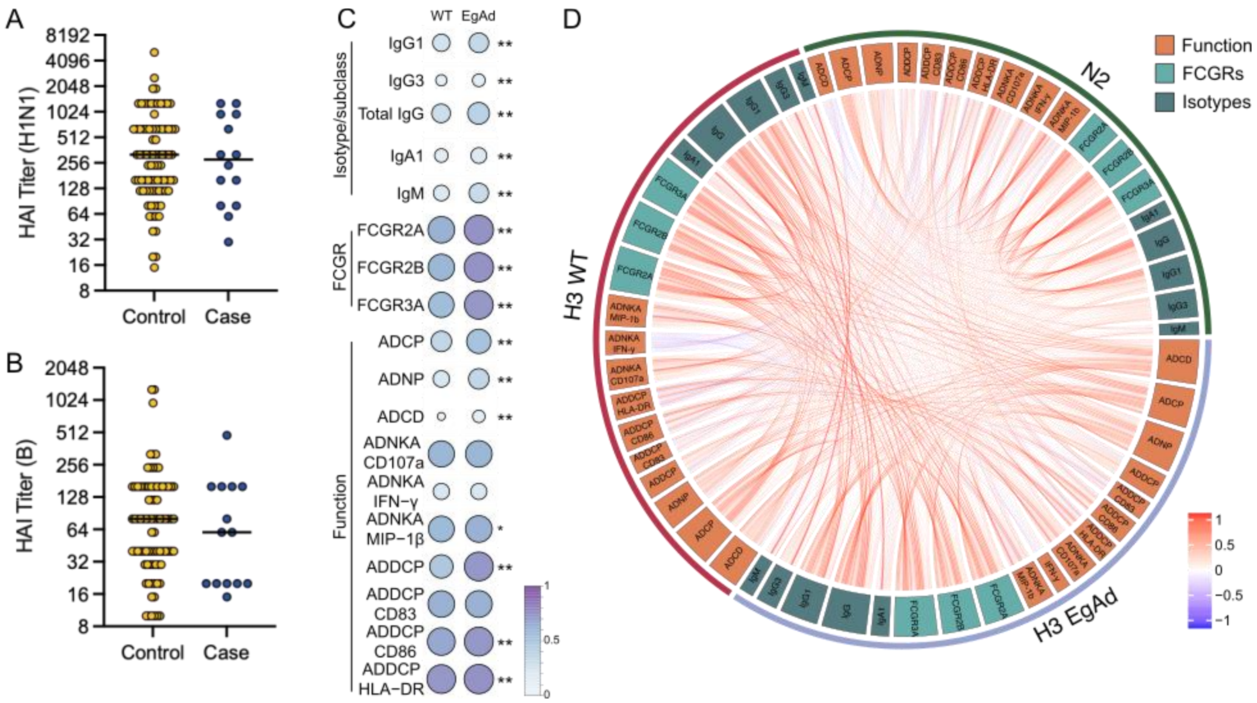

Figure S1. *Responses to vaccine and circulating strains are highly correlated but vaccine H3 responses have higher magnitude than circulating H3 responses.*

Serum samples form day 28 post-vaccination were analyzed for HAI, influenza-specific antibody levels, and influenza-specific antibody-mediated innate immune functionality. Dot plots show hemagglutination inhibition titers for controls (n = 86, yellow) who remained uninfected and cases (n = 14, blue) who became infected during the season against (A) H1N1 A/California/07/2009 and (B) B/Texas/6/2011. Differences between controls and cases were not significant. (C) Circles represent average antigen-specific antibody-dependent functional response, FCGR binding titer, or isotype titer across all participants (n=100) to both circulating H3 (H3 WT) and vaccine H3 (H3 EgAd). Sample values were z-score normalized across both antigens, then mean values represented in circle plots. Significance of difference between magnitude of response to each antigen is represented by stars, and was determined by two-sided Wilcoxon matched-pairs signed rank test with Bonferroni's correction. ADCP = antibody-dependent cellular phagocytosis, ADNP = antibody-dependent neutrophil phagocytosis, ADCD = antibody-dependent complement deposition, ADNKA = antibody-dependent NK cell activation, ADDCP = antibody-dependent Dendritic Cell phagocytosis. (D) Chord diagram shows correlations between antigen-specific antibody-dependent functional response, FCGR binding titer, or isotype titer across circulating H3 (H3 WT), vaccine H3 (H3 EgAd), and N2 antigens. Red chords represent positive Spearman R values and blue chords represent negative Spearman R values, with more intense color representing stronger correlation.

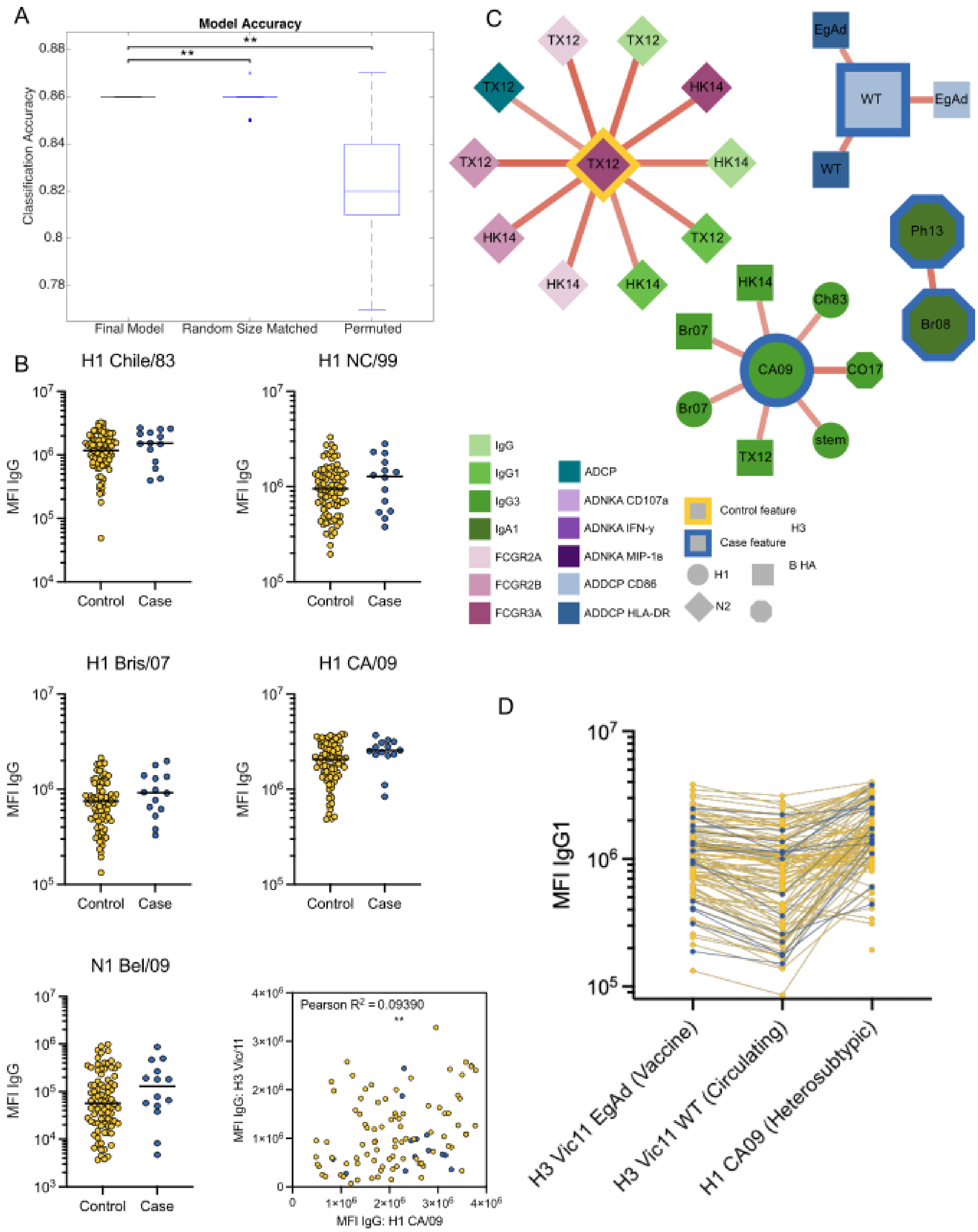

Figure S2. *Model significance, selected univariate data, and network of related features for LASSO-Elastic Net in Fig. 2.*
(A) The LASSO-Elastic Net model trained to distinguish between cases and controls was validated against random size matched and permuted label models. Accuracy over 100 permutations of each model was graphed with boxes showing quartiles, line showing median, and whiskers showing range. Outliers are graphed as dots. Significance tested by two-sided

Mann-Whitney U test, ** $p < 0.01$. (B) Total IgG levels specific for H1 and N1 antigens compared across controls and cases. Each dot represents the mean of two technical replicates for a single individual across controls (n = 86 individuals, yellow) and cases (n = 14 individuals, blue). Lines show median values and samples were compared by two-sided Mann-Whitney U tests. No comparisons were significant. Correlation plot shows Pearson Correlation Coefficient and significance (** $p = 0.0015$) for correlation between total IgG levels specific to vaccine strains H1 CA/09 and H3 Vic/11. (C) Networks show significant co-correlates, defined as spearman $R > 0.6$ and FDR-corrected $Q < 0.01$, of LASSO-Elastic Net selected features enriched in controls (highlighted in yellow) and cases (highlighted in blue). Abbreviations for influenza strains can be found in **Table S2**. (D) Comparison of IgG1 responses to vaccine (WT), circulating (EgAd), and heterosubtypic strain. IgG1 levels were determined by Luminex-based assay. Each dot represents mean of two technical replicates of one individual, with values for each individual connected by lines, across controls (n = 86 individuals) and cases (n = 14 individuals).

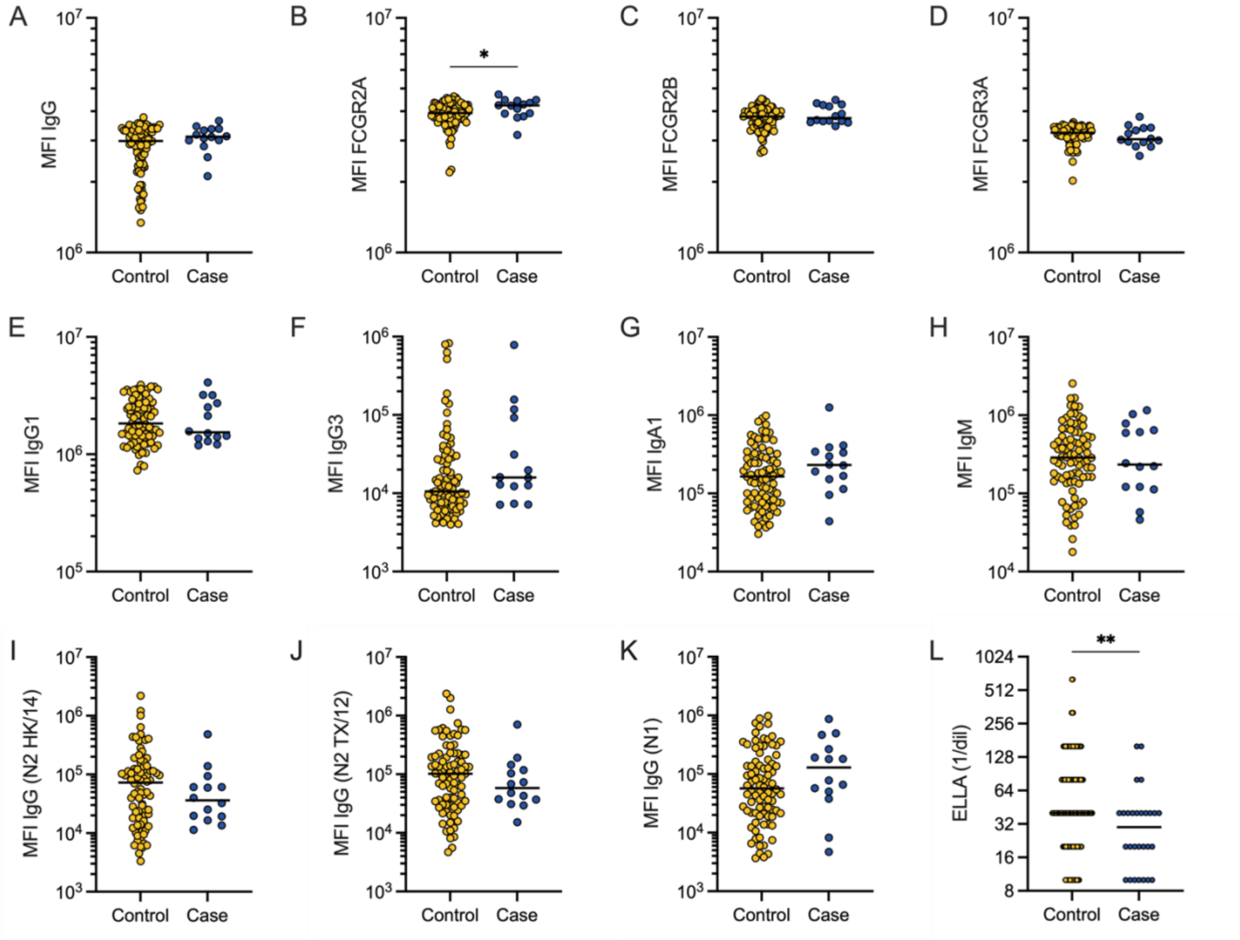

Figure S3. *H1 stem-specific and NA-specific responses do not consistently vary with infection status.*
(A-H) H1 stem-specific antibody isotype levels and FcR-binding levels as measured by Luminex bead-based assay for controls (n = 86 individuals, yellow) and cases (n = 14 individuals, blue). (I-L) Total IgG levels as measured by Luminex median fluorescence intensity (MFI) specific for (I) N2 A/Hong Kong/4801/2014, (J) N2 A/Texas/50/2012, and (K) N1 A/Belgium/145-MA/2009, and (L) ELLA H3N2 NAI results compared across controls and cases. Each dot represents mean of two technical replicates of one individual across controls (n = 86 individuals) and cases (n = 14 individuals). Lines show median values and samples were compared by two-sided Mann-Whitney U tests. * $p = 0.0479$, ** $p = 0.0063$.

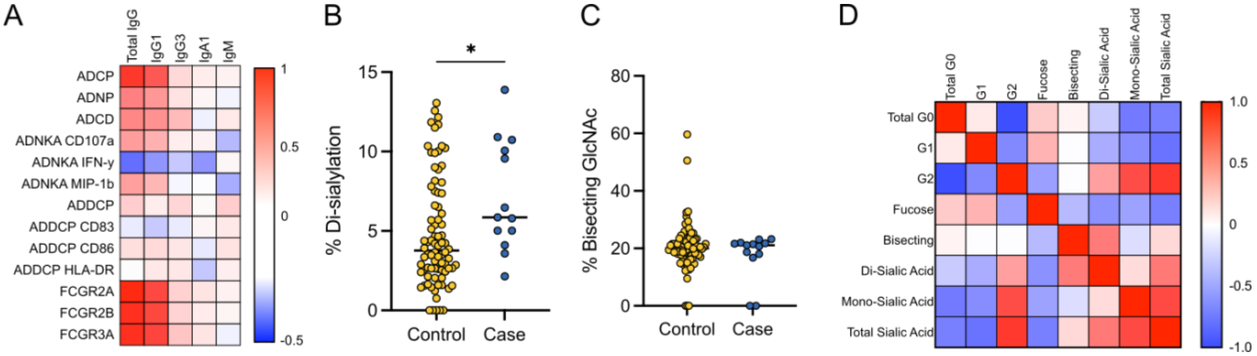

Figure S4. *NK functionality is driven primarily by IgG1.*
(A) Heat map shows Spearman correlations between antibody isotype levels and antibody-mediated functions/Fc receptor binding. All individuals (n=100) were included in this correlation analysis. ADCP = antibody-dependent cellular phagocytosis, ADNP = antibody-dependent neutrophil phagocytosis, ADCD = antibody-dependent complement deposition, ADNKA = antibody-dependent NK cell activation, ADDCP = antibody-dependent Dendritic Cell phagocytosis. (B) and (C) show levels of Fc di-sialylation (B) and bisecting GlcNAc (C) of H3 WT-specific antibodies in controls (n=86, yellow) and cases (n=14, blue) with lines at median values for each group. (B-C) Each dot represents a single measurement of one individual across controls (n = 86 individuals) and cases (n = 14 individuals). Significance tested by two-sided Mann-Whitney U test, * p = 0.0133, no marking = not significant. (D) Heat map shows Spearman correlations between different antibody Fc glycoforms. All individuals (n=100) were included in this correlation analysis.

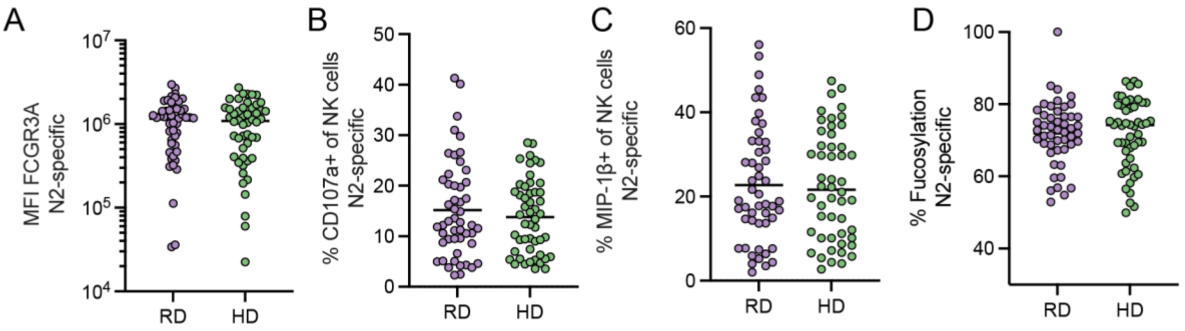

Figure S5. *High dose vaccination does not enhance antibody-mediated NK cell activation.* (A) shows H3-specific FCGR3A binding levels for regular (n=50, purple) and high dose vaccinees (n=50, green). (B) shows H3-specific NK cell CD107a expression for regular (n=50) and high dose (n=50) vaccinees. (C) shows H3-specific NK cell MIP-1b expression for regular (n=50) and high dose (n=50) vaccinees. (D) shows percentage of H3-specific antibodies that have fucosylated Fc glycans regular (n=50) and high dose (n=50) vaccinees. (A-C) Dots represent average of two technical replicates for a single individual. (A-D) Significance tested by two-sided Mann-Whitney U test, not significant.

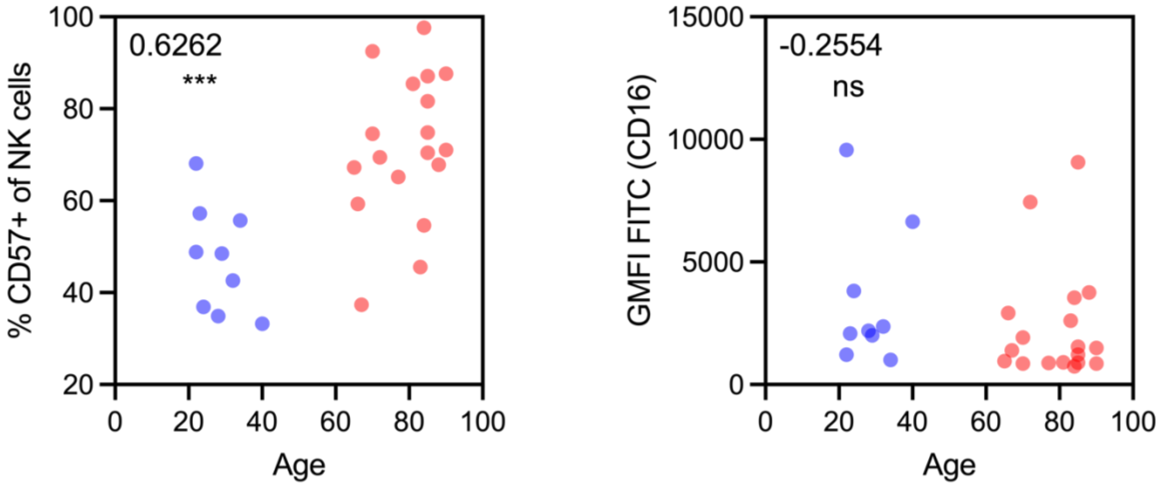

Figure S6. Dot plots show the percentage of NK cells positive for CD57 and expression level (geometric mean of fluorescence intensity, GMFI) of CD16 at baseline condition by age. Dots show individual subjects (<40 n=10 in blue, >65 n=19 in red). Correlation measured by Spearman's R, *** p = 0.0005.

	N (%)		N (%)
**Vaccination**		**Infection Status by Vaccination**	
Regular dose	50 (50%)	Influenza A	9 (18%)
		Influenza B	1 (2%)
High dose	50 (50%)	Influenza A	4 (8%)
		Influenza B	0 (0%)
**Infection**		**Vaccination Status by Infection**	
Control	86 (86%)	Regular dose	40 (47%)
		High dose	46 (53%)
Case	14 (14%)		
Influenza A (H3N2)	13 (13%)	Regular dose	9 (69%)
		High dose	4 (31%)
Influenza B	1 (1%)	Regular dose	1 (100%)
		High dose	0 (0%)

Table S1. *Study population summary.*
The table describes individuals included in this study based on vaccination and influenza outcome. The table further subsets individuals by looking at infection status dependent on vaccination and vaccination status dependent on infection.

	**Younger (<40)**	**Older (>65)**
Gender (study reported)	N (%)	N (%)
Male	3 (30%)	6 (32%)
Female	7 (70%)	13 (68%)
Most recent influenza vaccine		
Standard Dose	10 (100%)	12 (63%)
High Dose	0 (0%)	7 (37%)

Table S2. *Study population summary.*
The table describes individuals included in the PBMC study of older and younger adults based on sex (reported by study) and most recent influenza vaccination.

Influenza strain	Abbreviation	Ag-specific Ab isotype titers & FCR binding	Ag-specific Ab functions	Ag-specific Ab glycosylation
		FCGR2A<br>*FCGR2B*<br>*FCGR3A*<br>*IgA1*<br>*IgG1*<br>*IgG3*<br>*IgM*<br>*Total IgG*	*ADCP*<br>*ADNP*<br>*ADCD*<br>*ADNKA*<br>*ADDCP*	
H1N1 A/Chile/1/1983	H1Ch83	HA		
H1N1 A/New Caledonia/29/1999	H1NC99	HA		
H1N1 A/Brisbane/59/2007	H1Br07	HA		
H1N1 A/California/07/2009	H1CA09	HA		
H1N1 A/Belgium/145-MA/2009	H1Be09	NA		
H1 stabilized stem	H1 stem	HA		
H3N2 A/Panama/2007/1999	H3Pa99	HA		
H3N2 A/Brisbane/10/2007	H3Br07	HA		
H3N2 A/Victoria/361/2011	H3Vic	HA WT (circulating)<br>HA EgAd (Egg Adapted)	HA WT (circulating)<br>HA EgAd (Egg Adapted)	HA WT (circulating)
H3N2 A/Texas/50/2012	H3TX12	HA, NA	NA	NA
H3N2 A/Switzerland/9715293/2013	H3Sw13	HA		
H3N2 A/Hong Kong/4801/2014	H3HK14	HA, NA		
H3N2 A/Singapore/19/2016	H3Si16	HA		
B/Brisbane/60/2008	BBr08	HA		
B/Phuket/3073/2013	BPh13	HA		
B/Colorado/06/2017	BCO17	HA		

Table S3. *Antigens assayed in Systems Serology.*
Table describes the strains of Hemagglutinin (HA) and Neuraminidase (NA) antigens (Ag) used in each experiment, as well as the abbreviations used in figures where necessary for legibility.
