# Supplementary material for: Antibody-mediated NK cell activation as a correlate of immunity against influenza infection
Source: Nat Commun. 2023 Aug 24;14:5170. doi: 10.1038/s41467-023-40699-8 (PMC10449820; doi:10.1038/s41467-023-40699-8)
Supplement: Supplementary file 3 — Description of Additional Supplementary Files [file 41467_2023_40699_MOESM3_ESM.pdf]

Description of Additional Supplementary Files

File Name: Supplementary Software 1
Description: This .zip file contains the properly formatted source data and MATLAB code, including all custom dependencies, to create the analyses shown in Figure 2.
