# Supplementary material for: Antibody-mediated NK cell activation as a correlate of immunity against influenza infection
Source: Nat Commun. 2023 Aug 24;14:5170. doi: 10.1038/s41467-023-40699-8 (PMC10449820; doi:10.1038/s41467-023-40699-8)
Supplement: Supplementary file 5 — Reporting Summary [file 41467_2023_40699_MOESM5_ESM.pdf]

Corresponding author(s): Sridhar, Saranya
Alter, Galit

Last updated by author(s): 7/29/2023

Reporting Summary

Nature Portfolio wishes to improve the reproducibility of the work that we publish. This form provides structure for consistency and transparency in reporting. For further information on Nature Portfolio policies, see our Editorial Policies and the Editorial Policy Checklist.

Statistics

For all statistical analyses, confirm that the following items are present in the figure legend, table legend, main text, or Methods section.

n/a	Confirmed	
☐	☒	The exact sample size (*n*) for each experimental group/condition, given as a discrete number and unit of measurement
☐	☒	A statement on whether measurements were taken from distinct samples or whether the same sample was measured repeatedly
☐	☒	The statistical test(s) used AND whether they are one- or two-sided<br>*Only common tests should be described solely by name; describe more complex techniques in the Methods section.*
☐	☒	A description of all covariates tested
☐	☒	A description of any assumptions or corrections, such as tests of normality and adjustment for multiple comparisons
☐	☒	A full description of the statistical parameters including central tendency (e.g. means) or other basic estimates (e.g. regression coefficient) AND variation (e.g. standard deviation) or associated estimates of uncertainty (e.g. confidence intervals)
☐	☒	For null hypothesis testing, the test statistic (e.g. *F*, *t*, *r*) with confidence intervals, effect sizes, degrees of freedom and *P* value noted<br>*Give P values as exact values whenever suitable.*
☒	☐	For Bayesian analysis, information on the choice of priors and Markov chain Monte Carlo settings
☒	☐	For hierarchical and complex designs, identification of the appropriate level for tests and full reporting of outcomes
☐	☒	Estimates of effect sizes (e.g. Cohen's *d*, Pearson's *r*), indicating how they were calculated

Our web collection on statistics for biologists contains articles on many of the points above.

Software and code

Policy information about availability of computer code

Data collection	Data for this study was collected via flow cytometry using BD FACSDiva and Sartorius Forecyt (v7) and via capillary electrophoresis using ThermoFisher GlycanAssure v2 software.
Data analysis	Data in this study was analyzed using Flowjo v10, Graphpad Prism v9, R 3.5.1, SAS JMP Pro 14, Cytoscape v3.8.1, and Mathworks Matlab R2020a. R packages used were corrplot (https://cran.r-project.org/web/packages/corrplot/) and circlize (https://cran.r-project.org/web/packages/circlize/index.html). Matlab code used in this experiment has been included as a supplement to this submission, and is based on a previous publication: Ackerman, M. E. et al. Route of immunization defines multiple mechanisms of vaccine-mediated protection against SIV. Nat. Med. 24, 1590–1598 (2018).

For manuscripts utilizing custom algorithms or software that are central to the research but not yet described in published literature, software must be made available to editors and reviewers. We strongly encourage code deposition in a community repository (e.g. GitHub). See the Nature Portfolio guidelines for submitting code & software for further information.

Data

Policy information about availability of data

All manuscripts must include a data availability statement. This statement should provide the following information, where applicable:

- Accession codes, unique identifiers, or web links for publicly available datasets
- A description of any restrictions on data availability
- For clinical datasets or third party data, please ensure that the statement adheres to our policy

The data generated in this study are provided in the Supplementary Information/Source Data file.

Human research participants

Policy information about studies involving human research participants and Sex and Gender in Research.

Reporting on sex and gender	Neither sex nor gender information were available for older adult vaccination study samples.
Population characteristics	100 vaccinated older adult individuals were chosen for inclusion in this study. 50% of the individuals received standard dose vaccination and 50% received high dose vaccination. 86% remained uninfected throughout the study season while 14% became infected. The study population was ~95% white. In the parent study, 57% of participants were female. Most (73%) had received previous influenza vaccination. No genotypic information was collected. For further information, please see: DiazGranados, C. A. et al. Efficacy of High-Dose versus Standard-Dose Influenza Vaccine in Older Adults. N. Engl. J. Med. 371, 635–645 (2014).
Recruitment	Please see: DiazGranados, C. A. et al. Efficacy of High-Dose versus Standard-Dose Influenza Vaccine in Older Adults. N. Engl. J. Med. 371, 635–645 (2014).
Ethics oversight	Massachusetts General Hospital Institutional Review Board approved the current study.

Note that full information on the approval of the study protocol must also be provided in the manuscript.

Field-specific reporting

Please select the one below that is the best fit for your research. If you are not sure, read the appropriate sections before making your selection.

☒ Life sciences ☐ Behavioural & social sciences ☐ Ecological, evolutionary & environmental sciences

For a reference copy of the document with all sections, see nature.com/documents/nr-reporting-summary-flat.pdf

Life sciences study design

All studies must disclose on these points even when the disclosure is negative.

Sample size	Sample size for the vaccination cohort was a convenience sample of 100 individuals, determined by feasibility of data collection and sample availability.<br>Sample size for the differences between older and younger NK cells was chosen based on power calculations completed using previous experience of authors working with these cell types. See: Verschoor, C. P. et al. Key Determinants of Cell-Mediated Immune Responses: A Randomized Trial of High Dose Vs. Standard Dose Split-Virus Influenza Vaccine in Older Adults. Front. Aging. 2, (2021).
Data exclusions	Individual samples were excluded from Fc glycan analysis where identifiable capillary electrophoresis traces could not be identified. Individual wells of NK cell phenotyping (for the differences between older and younger NK cells) were excluded if <100 NK cells were captured. No other samples were excluded.
Replication	All systems serology measurements were successfully collected in duplicate and replicates were averaged to report final per-individual values. Where cell counts allowed, replicates were performed for older vs younger NK cell function (Figure 5) and averaged to report a final per-individual value.
Randomization	Randomization strategies for participants in the parent vaccine trial can be found here: DiazGranados, C. A. et al. Efficacy of High-Dose versus Standard-Dose Influenza Vaccine in Older Adults. N. Engl. J. Med. 371, 635–645 (2014).<br>No other experiments included differential treatments of separate samples.
Blinding	Investigators were blinded to the vaccine status and infection outcome of study participants during data collection and primary analysis. Group allocation was revealed once data collection and primary analysis was complete for further group-based analysis.

Reporting for specific materials, systems and methods

We require information from authors about some types of materials, experimental systems and methods used in many studies. Here, indicate whether each material, system or method listed is relevant to your study. If you are not sure if a list item applies to your research, read the appropriate section before selecting a response.

Materials & experimental systems

n/a	Involved in the study
[ ]	[x] Antibodies
[ ]	[x] Eukaryotic cell lines
[x]	[ ] Palaeontology and archaeology
[x]	[ ] Animals and other organisms
[ ]	[x] Clinical data
[x]	[ ] Dual use research of concern

Methods

n/a	Involved in the study
[x]	[ ] ChIP-seq
[ ]	[x] Flow cytometry
[x]	[ ] MRI-based neuroimaging

Antibodies

Antibodies used

Mouse anti-human IgG1 PE Southern Biotech Cat#9052-09
Mouse anti-human IgG3 PE Southern Biotech Cat#9210-09
Mouse anti-human IgA1 PE Southern Biotech Cat#9130-09
Mouse anti-human IgM PE Southern Biotech Cat#9020-09
Mouse anti-human CD66b Pacific Blue BioLegend Cat#305112
Goat anti-guinea pig C3 FITC MP Biomedical Cat#0855371
CD107a PE-Cy5 BD Biosciences Cat#555802
CD56 PE-Cy7 BD Biosciences Cat#557747
CD16 APC-Cy7 BD Biosciences Cat#557758
CD3 Pacific Blue BD Biosciences Cat#558117
MIP-1β PE BD Biosciences Cat#550078
IFN-ɣ FITC BD Biosciences Cat#340449
Biotin anti-human CD16 Antibody Biolegend Cat#302004
BUV395 Mouse Anti-Human CD3 BD Cat#563546
BD Horizon PE-CF594 Mouse Anti-Human CD56 BD Cat#562289
FITC anti-human CD16 Antibody Biolegend Cat#302006
PE/Cyanine7 anti-human CD158 Antibody Biolegend Cat#339512
PE/Cyanine7 anti-human CD158b Antibody Biolegend Cat#312610
Human NKG2A/CD159a Alexa Fluor 750-conjugated Antibody R&D Systems Cat#FAB1059S-100UG
Human NKG2C/CD159c APC-conjugated Antibody R&D Systems Cat#FAB138A-100
Brilliant Violet 605 anti-human CD314 Antibody Biolegend Cat#320832
Alexa Fluor 700 anti-human CD335 Antibody Biolegend Cat#331932
BV421 Mouse Anti-human CD57 BD Cat#563896
BV510 Mouse Anti-Human CD14 BD Cat#563079
BV650 Mouse Anti-Human CD69 BD Cat#563835
PE Mouse Anti-Human MIP-1b BD Cat#550078
Brilliant Violet 711 anti-human Perforin Antibody Biolegend Cat#308130

Validation

Validation relied on target specificity stated on the manufacturers websites, which is as follows.
Mouse anti-human IgG1 PE Southern Biotech Cat#9052-09: Based on ELISA and FLISA, antibody reacts to Human IgG1 Hinge; Mr 146 kDa.
Mouse anti-human IgG3 PE Southern Biotech Cat#9210-09: Based on ELISA and FLISA, antibody reacts to the Human IgG3 Hinge; Mr 170 kDa.
Mouse anti-human IgA1 PE Southern Biotech Cat#9130-09: Based on ELISA and FLISA, antibody reacts to the human IgA1 Fc; Mr 170 kDa.
Mouse anti-human IgM PE Southern Biotech Cat#9020-09: Based on ELISA, FLISA, and flow cytometry, antibody reacts to the human IgM; Mr 900 kDa.
Mouse anti-human CD66b Pacific Blue BioLegend Cat#305112: Based on immunofluorescent staining with flow cytometric analysis, antibody reacts to human CD66b.
Goat anti-guinea pig C3 FITC MP Biomedical Cat#0855371: The total protein is measured using the Biuret procedure, with bovine albumin as a standard. Antibody titer is standardized with an in-house control by immunoelectrophoresis. Each antiserum is tested for specificity at a minimum of 80mg/ml using immunoelectrophoresis. It shows reactivity to guinea pig complement C3; cross-reactivity to other species may exist. Antibody activity to other serum proteins is not present.
CD107a PE-Cy5 BD Biosciences Cat#555802: This antibody is routinely tested on the fixed and permeabilized Jurkat cells by flow cytometry with Cytofix/Cytoperm (Cat. No. 554714) for fixation and permeabilization.
CD56 PE-Cy7 BD Biosciences Cat#557747: This antibody is routinely tested by flow cytometry.
CD16 APC-Cy7 BD Biosciences Cat#557758: This antibody is routinely tested by flow cytometry.
CD3 Pacific Blue BD Biosciences Cat#558117: This antibody is routinely tested by flow cytometry.
MIP-1β PE BD Biosciences Cat#550078: For immunofluorescent staining and flow cytometric analysis, the D21-1351 antibody has been found useful to identify and enumerate MIP-1β producing cells within mixed cell populations. PE Mouse Anti-Human MIP-1β (Cat. No. 550078/561120) is especially suitable for these studies.
IFN-ɣ FITC BD Biosciences Cat#340449: To ensure consistently high-quality reagents, each lot of antibody is tested for

conformance with characteristics of a standard reagent.
Biotin anti-human CD16 Antibody Biolegend Cat#302004: Based on immunofluorescent staining with flow cytometric analysis, antibody reacts to human, cynomolgus, and rhesus CD16.
BUV395 Mouse Anti-Human CD3 BD Cat#563546: This antibody is routinely tested by flow cytometry.
BD Horizon PE-CF594 Mouse Anti-Human CD56 BD Cat#562289: This antibody is routinely tested by flow cytometry.
FITC anti-human CD16 Antibody Biolegend Cat#302006: Based on immunofluorescent staining with flow cytometric analysis, antibody reacts to human, cynomolgus, and rhesus CD16.
PE/Cyanine7 anti-human CD158 Antibody Biolegend Cat#339512: Based on immunofluorescent staining with flow cytometric analysis, antibody reacts to human CD158.
PE/Cyanine7 anti-human CD158b Antibody Biolegend Cat#312610: Based on immunofluorescent staining with flow cytometric analysis, antibody reacts to human CD158b.
Human NKG2A/CD159a Alexa Fluor 750-conjugated Antibody R&D Systems Cat#FAB1059S-100UG: Based on flow cytometry, this antibody recognizes the human NKG2A/CD94 heterodimer. It does not recognize the NKG2C/CD94 heterodimer or the CD94 homodimer.
Human NKG2C/CD159c APC-conjugated Antibody R&D Systems Cat#FAB138A-100: This antibody recognizes the human NKG2C/CD159c heterodimer with CD94 in flow cytometry. It does not cross-react with the human NKG2A/CD94 heterodimer or with the human CD94 homodimer.
Brilliant Violet 605 anti-human CD314 Antibody Biolegend Cat#320832: Based on immunofluorescent staining with flow cytometric analysis, antibody reacts to human CD314.
Alexa Fluor 700 anti-human CD335 Antibody Biolegend Cat#331932: Based on immunofluorescent staining with flow cytometric analysis, antibody reacts to human CD335.
BV421 Mouse Anti-human CD57 BD Cat#563896: This antibody is routinely tested by flow cytometry.
BV510 Mouse Anti-Human CD14 BD Cat#563079: This antibody is routinely tested by flow cytometry.
BV650 Mouse Anti-Human CD69 BD Cat#563835: This antibody is routinely tested by flow cytometry.
Brilliant Violet 711 anti-human Perforin Antibody Biolegend Cat#308130: Based on intracellular immunofluorescent staining with flow cytometric analysis, antibody reacts to human perforin.

No custom antibodies were used in these analyses.

Eukaryotic cell lines

Policy information about cell lines and Sex and Gender in Research

Cell line source(s): THP-1 cells were sourced from ATCC.

Authentication: THP-1 cells were not authenticated.

Mycoplasma contamination: Cell lines were not tested for mycoplasma contamination.

Commonly misidentified lines (See ICLAC register): No commonly misidentified cell lines were used in this study.

Clinical data

Policy information about clinical studies
All manuscripts should comply with the ICMJEguidelines for publication of clinical research and a completedCONSORT checklist must be included with all submissions.

Clinical trial registration: The parent study from which the vaccination study samples were drawn can be found at: NCT01427309.

Study protocol: Please see: DiazGranados, C. A. et al. Efficacy of High-Dose versus Standard-Dose Influenza Vaccine in Older Adults. N. Engl. J. Med. 371, 635–645 (2014).

Data collection: Please see: DiazGranados, C. A. et al. Efficacy of High-Dose versus Standard-Dose Influenza Vaccine in Older Adults. N. Engl. J. Med. 371, 635–645 (2014).

Outcomes: Please see: DiazGranados, C. A. et al. Efficacy of High-Dose versus Standard-Dose Influenza Vaccine in Older Adults. N. Engl. J. Med. 371, 635–645 (2014).

Flow Cytometry

Plots

Confirm that:

- [x] The axis labels state the marker and fluorochrome used (e.g. CD4-FITC).
- [x] The axis scales are clearly visible. Include numbers along axes only for bottom left plot of group (a 'group' is an analysis of identical markers).
- [x] All plots are contour plots with outliers or pseudocolor plots.
- [x] A numerical value for number of cells or percentage (with statistics) is provided.

Methodology

Sample preparation	PBMCs were isolated from whole blood draws and cryopreserved in 10% DMSO. PBMCs were thawed immediately prior to use.
Instrument	BD LSR Fortessa
Software	Data was collected using BD FACSDiva and analyzed using Flowjo 10.
Cell population abundance	Cell sorting was not performed as part of this study.
Gating strategy	For Figure 5: NK cells were identified as FSC/SSC, then by FSC-A/FSC-H for singlets, then as live cells, CD3-, CD14-, CD16+. The gating strategy is included in the figure.

☒ Tick this box to confirm that a figure exemplifying the gating strategy is provided in the Supplementary Information.
